# Gut microbiome signatures of nursing home residents carrying Enterobacteria producing extended-spectrum β-lactamases

**DOI:** 10.1186/s13756-020-00773-y

**Published:** 2020-07-14

**Authors:** Quentin Le Bastard, Guillaume Chapelet, Gabriel Birgand, Benjamin M. Hillmann, François Javaudin, Niki Hayatgheib, Céline Bourigault, Pascale Bemer, Laure De Decker, Eric Batard, Didier Lepelletier, Emmanuel Montassier

**Affiliations:** 1 MiHAR lab, Université de Nantes, 44000 Nantes, France; 2 Department of Emergency Medicine, CHU Nantes, Nantes University Hospital, 44000 Nantes, France; 3 Pole de gérontologie clinique, Nantes University Hospital, 44000 Nantes, France; 4 Regional Infection Control Centre, Pays de la Loire, Nantes, France; 5 Department of Computer Science and Engineering, University of Minnesota, Minneapolis, Minnesota USA; 6 Bacteriology and Infection Control Department, Nantes University Hospital, Nantes, France

**Keywords:** Microbiome, Antibiotic resistance, Enterobacteriaceae, Metabolome

## Abstract

**Background:**

The prevalence of extended beta-lactamase producing Enterobacteriaceae (ESBL-E) has been constantly increasing over the last few decades. These microorganisms that have acquired broad antibiotic resistance are now common human pathogens. Changes in the gut microbiome, induced by antibiotics or other drugs, enable expansion of these microorganisms, but the mechanisms are not yet fully understood.

**Objectives:**

The main objective was to identify specific bacteria and functional pathways and genes characterizing the gut microbiome of nursing home residents carrying ESBL-E, using metagenomics.

**Subjects and methods:**

We included 144 residents living in two different nursing homes. All fecal samples were screened for ESBL-E and gut microbiome was characterized using shallow shotgun metagenomic DNA sequencing.

**Results:**

Ten nursing home residents were colonized by ESBL-E, namely *Escherichia coli, Klebsiella pneumoniae* and *Enterobacter cloacae* species, and were compared to non-carriers. We found that ESBL-E carriers had an alteration in within-sample diversity. Using a bootstrap algorithm, we found that the gut microbiome of ESBL-E carriers was depleted in butyrate-producing species, enriched in succinate-producing species and enriched in pathways involved in intracellular pH homeostasis compared to non-carriers individuals. Several energy metabolism pathways were overrepresented in ESBL-E carriers suggesting a greater ability to metabolize multiple microbiota and mucus layer-derived nutrients.

**Conclusions:**

The gut microbiome of ESBL-E carriers in nursing homes harbors specific taxonomic and functional characteristics, conferring an environment that enables Enterobacteriaceae expansion. Here we describe new functional features associated with ESBL-E carriage that could help us to elucidate the complex interactions leading to colonization persistence in the human gut microbiota.

## Introduction

The spread of antimicrobial-resistant microorganisms is the main consequence of antibiotic overuse and misuse in human medicine and agricultural settings, leading to increased costs, treatment failures and mortality [1]. The rising prevalence of extended spectrum beta-lactamase producing Enterobacteriaceae (ESBL-E) worldwide is of particular concern [2]. Over the last decade in Europe, a significant increase in the prevalence of third-generation cephalosporin resistant *Escherichia coli* and *Klebsiella pneumoniae* was observed*,* from 0.1% in 1999 to 15% in 2017, and from 30.1% in 2011 to 37.2% in 2017, respectively [3]. The prevalence of ESBL-E infections varies from region to region, with alarming rates in the Asia Pacific region [4]. As Enterobacteriaceae are among the most frequently isolated pathogens in bacterial infections in Europe, the spread of ESBL-E strains will lead to increased use of carbapenem, raising fears about the diffusion of carbapenems-resistant micro-organisms [5]. Numerous evidence supports the hypothesis that antibiotic resistance genes could spread between pathogenic and commensal bacteria through horizontal transfers and that the human gut microbiota stands as the main reservoir of ESBL-E [6, 7].

Changes in the gut microbiota composition, mostly in anaerobes, induced by antibiotic or nonantibiotic drugs such as proton pump inhibitors or oral antidiabetic medication, enable colonization by exogenous resistant bacteria [8, 9]. The enrichment in some species, such as *Barnesiella* have been reported to provide a colonization resistance against vancomycin resistant *Enterococcus* but the interactions within bacterial communities conferring resistance to colonization are not yet fully understood [10, 11]. Two 16S rRNA based studies identified specific taxonomic profiles associated with resistance to ESBL-E colonization in humans and a recent experimental work highlighted the role of short chain fatty acids (SCFA) mediated inhibition of ESBL-E [12–14]. To date, such observations have not been confirmed in humans. The main objective of this study was to identify specific bacteria and functional pathways or genes characterizing the gut microbiome of nursing home residents carrying ESBL-E, using a metagenomics approach. The identification of these features could help us to understand the mechanisms involved in the establishment and persistence of a microbiological environment favorable to colonization by ESBL-E.

## Subjects and methods

### Subject recruitment and sample collection

The following protocol was approved by the Nantes University institutional review board (IRB). This study was part of a point prevalence survey of ESBL-E and carbapenemase-producing Enterobacteriaceae gastrointestinal carriage conducted from July to August 2016 [15]. The study population consisted of 144 elderly residents from two public nursing homes (312 residents) in Nantes (Western France). Residents were enrolled according to their (or their surrogate) ability to provide written consent. Age, morphological data, comorbidities, current and previous medications including antibiotics, were collected from medical records.

Fecal samples were collected by the nursing staff by swabbing freshly emitted stools (Copan or FecalSwab, Copan Diagnostics, Murrieta, CA, USA) in each participant for microbiological identification of ESBL-E (see Supplementary materials for laboratory methods). Samples were addressed and managed in the laboratory of Bacteriology, Nantes University Hospital, for identification of ESBL, according to CLSI guidelines. Samples were vortexed and cultured for 24 h on specific chromogenic agar culture medium (ESBL-chromID, Biomérieux, France). Colonies were identified using mass spectrometry (MALDI-TOF MS method, Bruker Daltonics, Germany). ESBL-E phenotypes were confirmed by detection on disc diffusion tests (MASTDISCSTM D72C ID ESBL test, Mast Diagnostics, Merseyside, UK). Samples for metagenomic analysis were immediately stored at 4 °C in RNA later (Fisher scientific, Waltham, MA, USA) during 24 h and then at − 80 °C until sequencing.

### Shotgun metagenomics analysis

DNA extraction, amplification, and sequencing were performed at the University of Minnesota Genomics Center (UMGC). DNA was extracted from frozen fecal samples using the MO BIO PowerSoil DNA Isolation Kit (MO BIO, Carlsbad, CA, USA) and quantified using the Quant-iT PicoGreen dsDNA Assay kit (Fisher scientific, Waltham, MA, USA). A shallow shotgun sequencing of total stool DNA was applied using Illumina HiSeq platform. Paired-end sequencing was performed using 2 × 250 base-pair reads following manufacturer recommendations. Libraries were prepared using Illumina barcodes (TruSeq DNA Sample Prep kit) and KAPA biosystems reagents (KAPA Library Preparation kit). The resulting DNA libraries were amplified by PCR using KAPA HiFi polymerase (Roche, Bâle, Switzerland) [16]. Reads were aligned up to species level against the comprehensive NCBI RefSeq database release 87 [17] using BURST aligner (v0.99.7) with a threshold of 97% identity [18, 19]. All matched bacterial species were included. Functional annotations were obtained using the HUMAnN2 [20] pipeline (v0.11.2) against KEGG bacteria and archaea genome database (v56). Statistical analysis were performed in the R (version 3.4.1) environment.

Within-samples taxonomic and functional biodiversity (alpha diversity) were measured using the QIIME2 (version 2018.4) diversity script after multiple rarefactions at species level (210,696 sequences/samples) [21]. Relative abundance were computed using the QIIME2 feature-table script. CAZyme annotations were obtained using Diamond (version v0.9.24.125, default parameters -k 25 and -e 0.001) and the CAZy database (release 07/26/2019) [22, 23]. We compared Chao1 and unique observed-species between groups using a nonparametric two-sample mean difference test with 999 Monte Carlo permutations. We quantified taxonomic and functional biodiversity differences between samples (beta-diversity) by computing Bray Curtis distances with the *vegan* R package (version 2.5–1) between all pairs of samples and compared groups using ANOSIM with 999 Monte Carlo permutations. Then, we identified differences in taxonomic and functional items abundance between groups by estimating log2-fold change with the likelihood ratio test included in the DESeq2 package (v1.18.1) [24]. We used a model fitting a local regression of log dispersion over log base mean with a negative binomial distribution. *P*-values were adjusted for multiple testing using the False Discovery Rate (FDR) (Benjamini-Hochberg adjustment).

We used a bootstrap algorithm with 200 iterations to compare ESBL-E carriers and non-carriers. In detail, we randomly selected 200 times 40 ESBL-E non-carriers samples that were compared to the ESBL-E carriers (*n* = 10) with DESeq2. All taxonomic and functional items bootstrapped mean log2-fold changes with adjusted *p* values < 0.10 were considered as statistically significant and plotted. We considered a robust signature, an item that was significantly different between 2 groups in at least 70% of all repeated procedures [25, 26].

The associations of individual species to collected covariates were assessed using MaAsLin (v0.0.4) with default settings in R environment [27]. In each analysis, the false discovery rate was controlled at FDR corrected *p* value 0.1 using the R-package Q-value (v3.10). (See supplemental methods for more details).

## Results

### Study population

We enrolled 144 of the 312 residents of the two nursing home. Ten (6.9%) of them were confirmed to carry ESBL-E. Microbiological analysis identified seven *Escherichia coli*, two *Enterobacter cloacae* and one *Klebsiella pneumoniae.* The relative abundance of these species was not significantly different between carriers and non-carriers (Mann-Whitney U test, ESBL-E carriers versus non-carriers for *E. coli, E. cloacae and K. pneumoniae* relative abundances, all *p* values > 0.05) (Supplementary Figure S1). There were no significant differences in baseline characteristics between carriers and non-carrier subjects, including antibiotic exposure (Table 1). Prevalence of ESBL-E carriage were not different between the two nursing homes (Fisher’s exact test, *p* value > 0,05). Among ESBL-E carriers, four were exposed to broad spectrum penicillin during 7 days. As all residents were housed in two nursing homes from the same University hospital, diet, hygiene and care was performed to a standardized practice by the same medical and nursing staff and meals were prepared in the same facility.

**Table 1:** Baseline characteristics of ESBL-E carriers and non-carriers among residents of the two nursing homes. Univariate analysis were performed using Wilcoxon signed rank test

	No. of residents (%)		***P*** value
Baseline Characteristics	ESBL-E carriers (n = 10)	Non carriers (***n*** = 134)	
Sex (females)	7 (70)	90 (67)	0.8
Age, mean (SD)	85.6 (±5.82)	86.13 (±6.61)	0.53
Dementia	7 (70)	78 (58)	0.48
Parkinson disease	0 (0)	9 (7)	0.4
Diabete	0 (0)	30 (22)	0.095
Peptic ulcer disease	1 (10)	13 (10)	0.98
Chronical kidney disease	1 (10)	14 (10)	0.96
Long term stay (> 24 months)	5 (50)	94 (70)	0.188
**Medications during past 3 months**			
Oral antidiabetic medication	1 (10)	10 (7)	0.78
Anti-acid medication	4 (40)	26 (19)	0.124
Broad spectum pennicilin	4 (40)	41 (31)	0.54
Anxiolytic medication	7 (70)	98 (73)	0.82
Antidepressant medication	3 (30)	75 (56)	0.11
Antiepileptic medication	1 (10	24 (18)	0.53
Proton-pump inhibitors	1 (10)	5 (4)	0.35

### Diversity of the gut microbiome is lower in ESBL-E carriers

We obtained the mean 308,696 sequences per samples, providing enough depth to reach species-level [16].

**Fig. 1:**
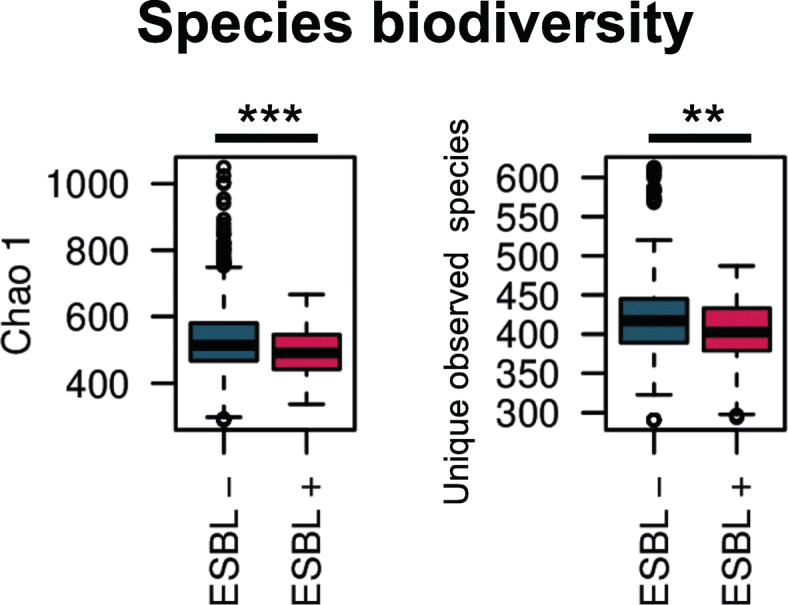
Alpha-diversity indices in gut microbiomes of extended spectrum beta-lactamase producing Enterobacteriaceae (ESBL-E) carriers (ESBL+) and non-carriers (ESBL-), based on species. Whiskers in the boxplot represent the range of minimum and maximum alpha diversity values within a population, excluding outliers. Monte-Carlo permutation t-test: * *p* value < 0.05; ** *p* value < 0.01; and *** *p* value < 0.001. Boxplots denote top quartile, median, and bottom quartile. (ESBL-E) carriers had significantly lower microbial richness compared with non-carriers, using Chao1 and unique observed species (*p* value < 0,001 and *p* value < 0,01, respectively)

Comparison of within-samples species diversity (alpha-diversity), using Chao1 and unique observed species indexes, showed a less diverse gut microbiome in ESBL-E carriers (Mann-Whitney U test, ESBL-E carriers versus non-carriers, Chao1 and unique observed species, *p* value < 0.001 and *p* value < 0.01, respectively, Fig. 1). Principal-coordinates analysis (PCoA) of Bray Curtis distances revealed that carriers and non-carriers also harbored a slight but significantly distinct gut microbial compositions. The R value, close to 0 suggests the existence of an important overlap area between the 2 groups (analysis of similarities, ANOSIM, R = 0.177, *p* value = 0.048, Supplementary Figure S2). Carriers and non-carriers were not clustered according to their functional architecture (ANOSIM, R = 0.1, *p* value = 0.15), but we observed a trend in increased functional biodiversity in carriers (2,129,865 sequences/samples, Mann-Whitney U test, ESBL-E carriers versus non-carriers, Chao1 and unique observed species, *p* value < 0.01 and *p* value < 0.175, respectively, Supplementary Figure S3). Importantly, we did not find any significant association between other covariables and interindividual microbial distance, including sex, age, length of stay, comorbidities and medications.

### ESBL-E carriers gut microbiome is enriched in *Bacteroides* and *Prevotella*

**Fig. 2:**
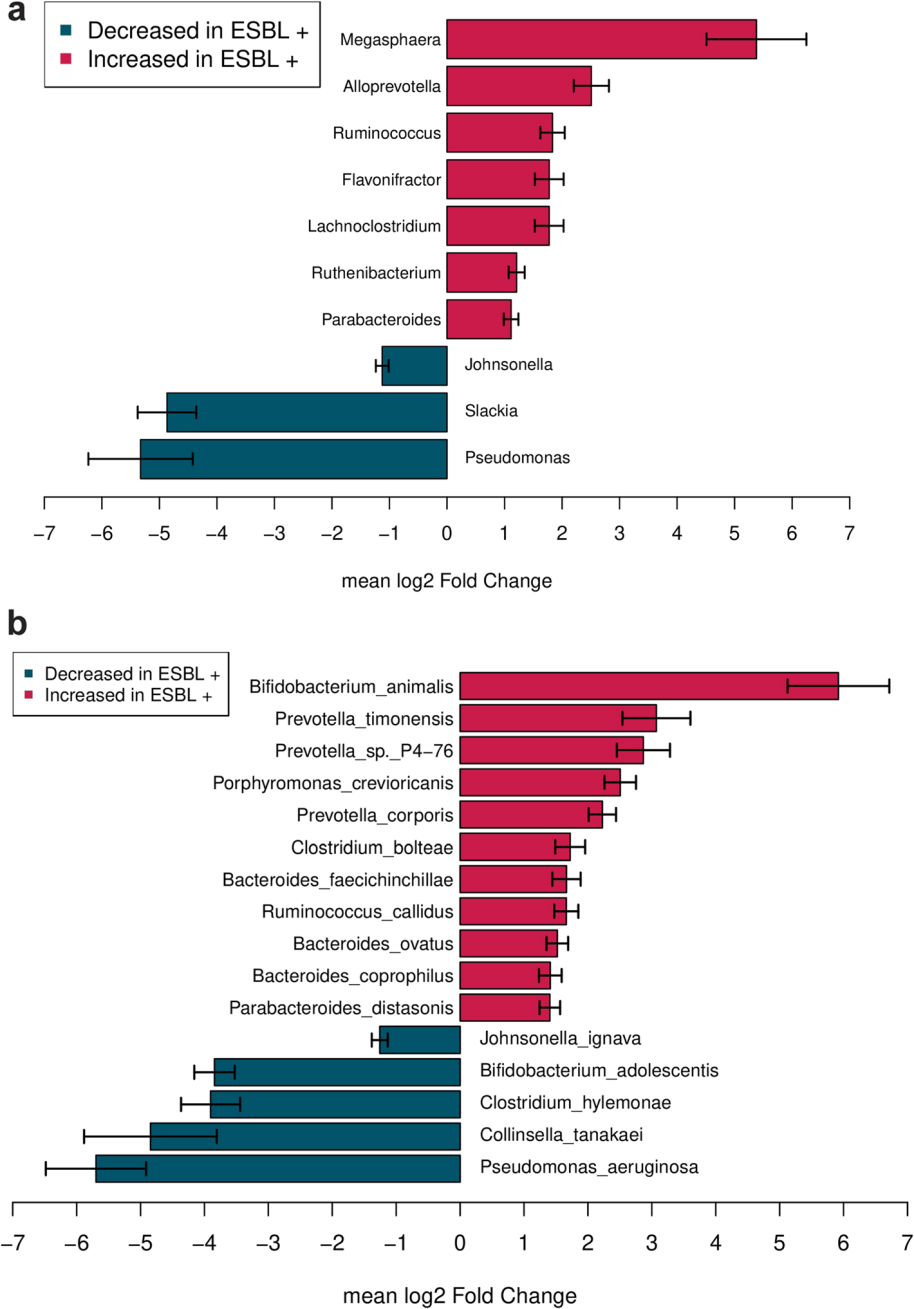
**a** Microbes that differentiate Enterobacteriaceae (ESBL-E) carriers and non-carriers at genus level, using DESeq2 with bootstrap iterations. The bootstrap model consists in 200 random selections of non-carriers (*n* = 40). We represented the genera that are significantly different between ESBL-E carriers and non-carriers in more than 70% of the iterations, with a FDR corrected *p* value < 0.10. Genera with increased abundance in ESBL-E carriers are represented in red, and genera with decreased abundance are represented in blue, **b** Microbes that differentiate Enterobacteriaceae (ESBL-E) carriers (ESBL+) and non-carriers (ESBL-) at species level, using DESeq2 with bootstrap iterations. The bootstrap model consists in 200 random selections of non-carriers (*n* = 40). We represented the species that are significantly different between ESBL-E carriers and non-carriers in more than 70% of the iterations, with a FDR corrected *p* value < 0.10 . Species with increased abundance in ESBL-E carriers are represented in red, and species with decreased abundance are represented in blue

To identify a taxonomic signature associated with ESBL-E carriage, we compared bacterial communities of carriers and non-carriers at genus and species levels using DESeq2 with bootstrap iterations. The great majority of genera overrepresented in the gut microbiome of ESBL-E carriers mapped to *Bacteroidales* spp. and *Clostridiales* spp. orders (respectively 4 and 2 of 9). In particular, samples from carriers showed a more than 2 mean log2fold higher proportion of *Megasphaera* spp., *Alloprevotella* spp. (FDR corrected *p* value < 0.10, in 70% or more bootstrapping iterations). Less frequently, *Prevotella* spp. and *Bacteroides* spp. appeared increased in ESBL-E carriers (FDR corrected *p* value < 0.10, in 60% or more bootstrapping iterations). The representation of *Pseudomonas* spp. and *Johnsonella* spp. genera appeared strongly decreased with a mean log2fold change below − 4 (FDR corrected *p* value < 0.10, in 70 and 60% or more bootstrapping iterations respectively) (Fig. 2a, Supplementary Table S1).

More precisely, bacterial species composing the community in ESBL-E carriers were significantly enriched in 8 species. *Bifidobacterium animalis* showed a strongly increased abundance of 6 mean log2fold change and three *Prevotella* species were increased over 2 mean log2fold change (*P. corporis, P. timonensis* and *P sp. P4–*76). Species belonging to *Bacteroides* spp. genera represented a large proportion of the overrepresented individual bacteria in these residents (*B. faecichinchillae, B. coprophilus, B. ovatus*). The lower proportion of *Johnsonella* spp. genera in ESBL-E carriers was mostly represented by a lower abundance of *J. ignava* and *Pseudomonas* spp. genera by *Pseudomonas aeruginosa* (FDR corrected *p* value < 0.10, in at least 70 and 60% or more bootstrapping iterations respectively)*.* Three individual bacterial species showed a strongly decreased abundance below − 3 mean log2fold change, namely *Clostridium hylemona* and *Collinsella tanakaei* (FDR corrected *p* value *<* 0.10, in at least 70% or more bootstrapping iterations) and less frequently *Bifidobacterium adolescentis (*FDR corrected *p* value < 0.10, in at least 60% or more bootstrapping iterations) (Fig. 2b, Supplementary Table S2).

As many parameters may influence the composition of the gut microbiome, we used the MaAslin pipeline to test for association between baseline characteristics and taxonomical composition of the gut microbiome of all residents. We did not find any significant association between age, sex, length of stay, medications, comorbidities and taxonomical composition of the gut microbiome.

### The gut microbiome of ESBL-E carriers is enriched in carbohydrate metabolism pathway genes

**Fig. 3:**
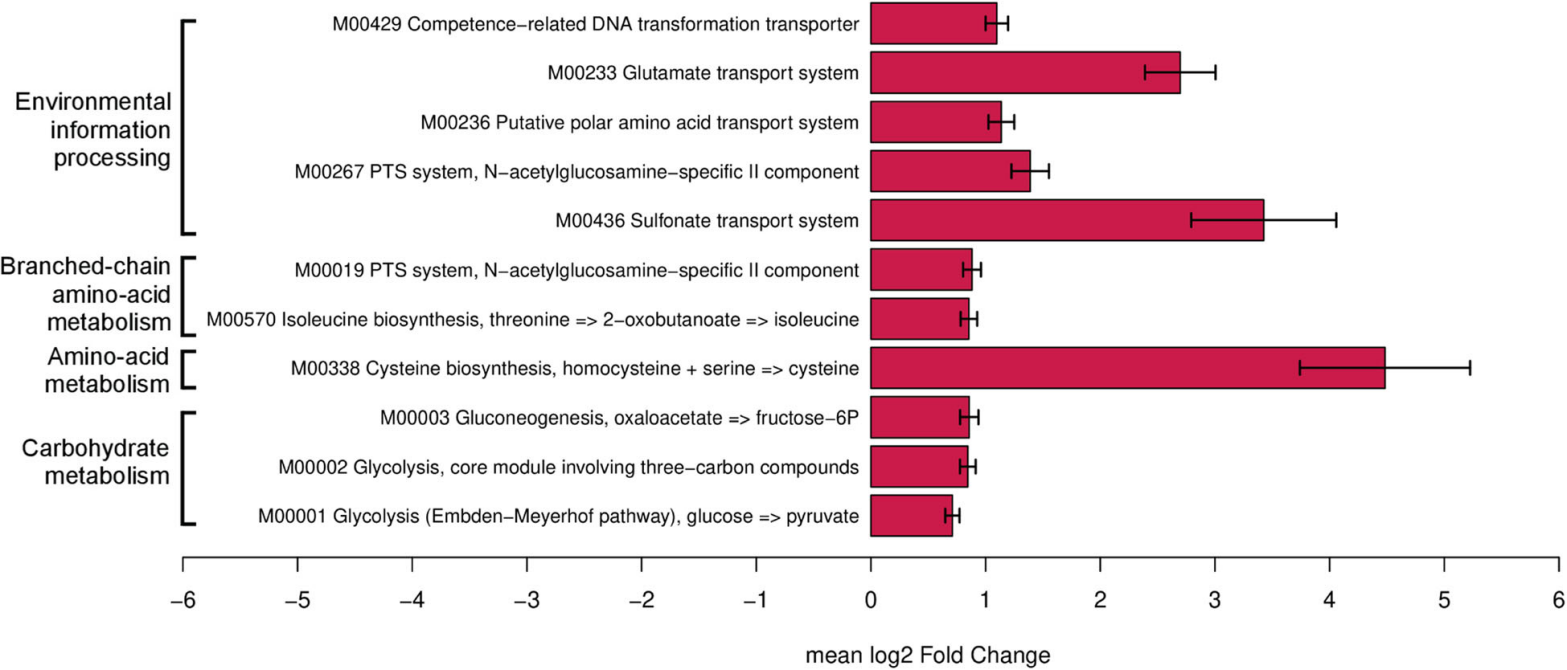
Modules that differentiate Enterobacteriaceae (ESBL-E) carriers and non-carriers, using DESeq2 with bootstrap iterations. The bootstrap model consists in 200 random selections of non-carriers (n = 40). We represented the modules that are significantly different between ESBL-E carriers and non-carriers in more than 70% of the iterations, with a FDR corrected *p* value < 0.1. Functional modules with increased abundance in ESBL-E carriers are represented in red, and functional modules with decreased abundance are represented in blue

The analysis of functional modules harbored by the gut microbiome showed robust differences in relative abundance of 8 KEGG modules between ESBL-E carriers and non-carriers (FDR corrected *p* value *<* 0.10, in 70% or more bootstrapping iterations) (Fig. 3, Supplementary Table S3)*.* All functional pathways identified were overrepresented in ESBL-E carriers and were involved in carbohydrate metabolism, amino-acid and branched-chain amino acid metabolism and environmental information processing. Among them, KEGG modules involved in cysteine biosynthesis (M00338), glutamate transport (M00233), sulfonate transport (M00436), N-acetylglucosamine biosynthesis (M00267 and M00019) and isoleucine biosynthesis (M00570) were the most frequently significant. We identified a total of 50 KEGG orthologies (KOs) with modified relative abundance between carriers and non-carriers, which were all overrepresented in ESBL-E carriers gut microbiome (FDR corrected *p* value *<* 0.10 in 70% or more bootstrapping iterations) (Supplementary Figure S4 and supplementary Table S4). Finally, to further investigate carbohydrate metabolism, we searched for a signature in CAZymes composition. Nine CAZymes had a modified relative abundance between carriers and non-carriers, all overrepresented in ESBL-E carriers gut microbiome (FDR corrected *p* value *<* 0.10 in 70% or more bootstrapping iterations) (Supplementary figure and supplementary Table S5)*.*

## Discussion

The colonization of the human gut microbiome by antibiotic-resistant Enterobacteriaceae strains is of peculiar concern in clinical practice, as it exposes to the risk of spread within the community, increasing the subsequent risk of disseminated infections, including bacteremia [28]. Here, we focused on describing the taxonomical and functional signature of ESBL-E carriers gut microbiome. When compared to non-carriers, the community is primarily characterized by a decreased abundance in SCFAs-producing bacteria, an increased ability to produce succinate, and an increased ability to metabolize carbohydrates derived from the mucus layer. Our understanding of the interactions contributing to colonization by resistant germs within the microbiota is limited. Our results confirm in humans the existence of mechanisms observed in vitro that could be future therapeutic targets for preventive or curative strategies. We did not identify any other parameters influencing the composition of the gut microbiome in this cohort. To our knowledge, this is the first study aimed to explore the specific patterns of the gut microbiome associated with ESBL-E carriage using shotgun metagenomic sequencing, providing a deeper description of bacterial communities and functional pathways than previous works in the field.

**Fig. 4:**
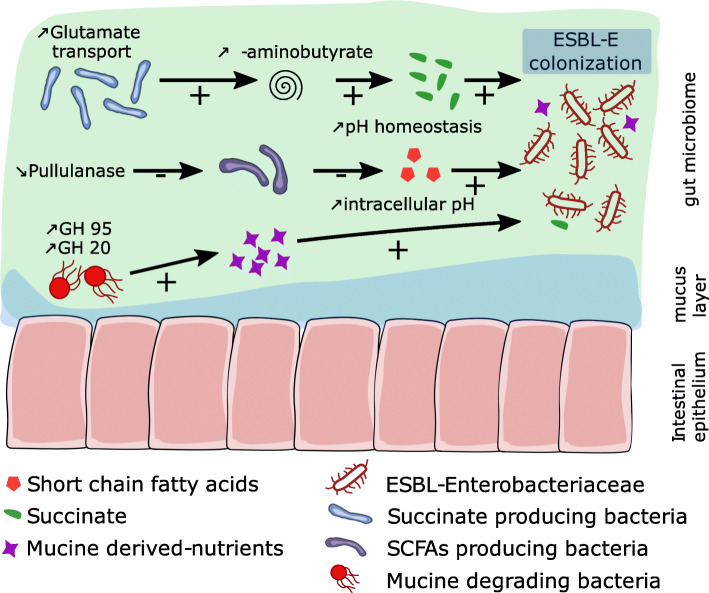
Proposed mechanisms that may play a role in the expansion of antimicrobial-resistant Enterobacteriaceae in the human gut microbiome

Overall, we found that our results are consistent with previous experimental observations and highlight several mechanisms that may play a key role in the expansion of antimicrobial-resistant in the human gut microbiome (Fig. 4). Among them, SCFAs production seems to be the most crucial function of the gut microbiota in terms of providing colonization resistance against ESBL-E [14]. In this cohort, we observed that the gut microbiome of ESBLE-E carriers was characterized by a lower abundance of SCFAs producing bacteria. A large proportion of the bacteria we found depleted in the gut microbiome of ESBL-E carriers, namely *Clostridium hylemonae, Collinsella tanakaei, Johnsonella ignava,* and *Bifidobacterium adolescentis* have been identified as being directly involved in SCFAs production such as acetate, propionate, and butyrate. This supports the hypothesis that a depletion in SCFAs producing species may be associated with ESBL-E colonization and persistence in the gut [29–31]. Moreover, the only under-represented metabolic feature in ESBL-E carriers was pullulanase (K01200), a glucanase that degrades pullulan, which has been reported to stimulate butyrate production by promoting the growth of *Bifidobacteria* species [32]. A recent experimental work highlighted that the maintenance of an acidic environment coupled with production of high concentration of SCFAs by the colonic microbiota promote the clearance of EBLS-E [14]. The authors demonstrate that SCFAs can directly trigger intracellular acidification of ESBL-E to counter the competitive edge that O_2_ and NO_3_ respiration confer upon Enterobacteriaceae during expansion. Here, the most significantly overrepresented species in ESBL-E carriers was *Bifidobacterium adolescentis*. This bacterium was also found increased in the gut microbiome of travelers who eradicate ESBL-E colonization when compared to travelers who did not suggesting it could play a key role in colonization clearance of antibiotic resistant bacteria. In the same way, travelers who did not cleared ESBL-E colonization harbored a significantly higher proportion of *Bacteroides sp.* in their gut microbiome such as ESBL-E carriers in our study, suggesting a role for this genus in colonization persistence of antibiotic resistant bacteria [33].

In our analysis of the functional profile of ESBL-E carriers, we found evidences suggesting an increased capacity of succinate production. There were a greater abundance of gene that function in the transport of glutamate (M00233, K10008, K10007 and K10006) in ESBL-E carriers suggesting an increased glutamate metabolism. Glutamate is involved in the biosynthesis of several proteins and mostly metabolized via the GABA shunt pathway. More specifically, the production of γ-aminobutyrate through a decarboxylation reaction of glutamate consumes protons which are subsequently removed from the environment. The production of succinate from glutamate by Glutamate Decarboxylase has been reported to be implicated in bacterial acid tolerance by facilitating the homeostasis of intracellular pH [34]. These observations were consistent with the enrichment of the intestinal microbiome of ESBL-E carriers with bacteria of the genera Prevotella and Bacteroides. Both are known to produce succinate, suggesting that their overrepresentation in the microbiota could lead to greater availability of succinate [34, 35]. Some bacterial pathogens have been described to metabolize microbiota-derived succinate for their own benefit. In *Salmonella typhimurium*, succinate metabolism contributes to efficient colonization of the intestinal lumen, and in *Clostridium difficile* it promotes the onset of infection after antibiotic treatment [36, 37]. Overall, these data suggest that modulations of the gut microbiome leading to increased succinate production and a reduced SCFAs availability could provide a favorable environment for ESBL-E colonization.

Our data also show that the gut microbiome of ESBL-E carriers expresses a greater diversity of energy metabolism pathways with an overrepresentation of gluconeogenesis (M00003), glycolysis (M00001, M00002) and pentose-phosphate pathway (K07404). The greater abundance of modules that function in amino-acid metabolism, especially isoleucine and methionine (M00570, M00019, M00338) and gluconeogenesis (M00003) suggests an intense energy metabolism activity involving alternative pathways that use amino-acids instead of carbohydrates to generate glucose. This type of mechanism has been observed in urinary tract infections due to *Escherichia coli* [38]. The increased diversity of genes operating in energy metabolic pathways may reflect a greater ability to metabolize multiple microbiota-derived nutrients. This could provide a growth facility for certain species such as Enterobacteriaceae and promote colonization as previously described with gluconeogenesis for the maintenance of *Escherichia coli* O157:H7 in the microbiota [39]. We observed an increased abundance of glycoside hydrolase genes in the microbiome of ESBL-E carriers. In particular, the overrepresentation of the glycoside hydrolase genes GH 95 and GH 20 suggests that the microbiome of ESBL-E carriers exhibits increased mucin degradation capacity. Mucin degradation results in increased availability of host-derived sugar, such as fucose, which has been shown to facilitate post-antibiotic expansion of enteric pathogens [40, 41]. Thus, the gut signature of ESBL-E carriers is characterized by a greater ability to metabolize multiple microbiota and mucus layer derived carbohydrates. This suggests that colonization is enabled by the establishment of a favorable nutrient environment.

Finally, metabolic pathways suspected to be part of the bacterial mechanisms involved in colonization were also overrepresented in ESBL-E carriers. Among them, we identified an increased abundance of pathways involved in sulfur cycling (M00436, K15553, K15555), supposed to increase bacterial resistance to antibiotics and to protect them from reactive oxygen species [42].

### Limitations

Our study has several limitations, the main one being the low prevalence of ESBL-E colonization in our cohort, the reason why we used bootstrap iterations. This has also limited the statistical power when comparing of baseline characteristics of colonized and non-colonized residents. Second, although subjects included in our cohort were exposed to controlled medication and received the same dietary, the same hygiene and care support, all the parameters that may affect gut microbiome composition, such as visits by outside individuals, were not controlled. Third, this study only provides information on the characteristics of the gut microbiome allowing for persistence of colonization but neither explains the initial changes that occurs nor the mechanisms leading to the acquisition of these resistant strains, as longitudinal design could have done. Also, because this work is based on DNA sequence reads analysis, it only provides informations about metabolic coding capacities but do not prejudge that these sequences are transcribed into actual functions. The use of shallow shotgun provides an information about species communities and functional capacities but is not as accurate as deep-shotgun. Thus, poorly represented species or functional genes may have been missed with this strategy. Further investigations, including transcriptomic, metabolomic analysis and multiple time points are needed to confirm the upregulation of these metabolic pathways and the increased availability of succinate in the gut lumen of ESBL-E carriers.

## Conclusions

In this work we described the gut-microbiome signature of ESBL-E carriers. Our observations highlight several mechanisms involved in the colonization of the human gut microbiota by ESBL-E in nursing home residents. This includes a modification in acidity regulation with a decreased SCFAs production and a greater ability to regulate intracellular pH through an increased succinate availability providing an electron-donor for respiratory chain. The greater availability of microbiota-derived nutrients derived from the mucus layer degradation, combined with increased energy metabolism pathways, could provide a growth facility for ESBL-E and promote colonization. Overall, these mechanisms confer an environment enabling the establishment and survival of Enterobacteriaceae in the gut. These observations, especially the role of succinate, require further investigation, including metabolomic, and could help us to elucidate the complex interactions leading to colonization persistence in the human gut microbiota. Future works should focus on setting up a metabolomics pilot study, including multiple time point samplings, to investigate the role of succinate. Tailored shifts in the gut microbiota based on the administration of complex consortia of bacteria able to increase SCFAs production and to decrease succinate availability might prevent the colonization and contain the spread of antimicrobial-resistant microorganisms in humans.

## Supplementary information

**Additional file 1.** Supplementary methods and figures.

**Additional file 2. Supplementary Table 1.** Genus that differentiate carriers and non-carriers using DESeq2 with bootstrap iterations.

**Additional file 3. Supplementary Table 2.** Species that differentiate carriers and non-carriers using DESeq2 with bootstrap iterations.

**Additional file 4. Supplementary Table 3.** KEGG modules that differentiate carriers and non-carriers using DESeq2 with bootstrap iterations.

**Additional file 5. Supplementary Table 4.** KEGG KO that differentiate carriers and non-carriers using DESeq2 with bootstrap iterations.

**Additional file 6. Supplementary Table 5.** CAZyme that differentiate carriers and non-carriers using DESeq2 with bootstrap iterations.

## Data Availability

The datasets generated during the current study are available in the NCBI Sequence Read Archive under BioProject ID PRJNA531316.

## Abbreviations

ESBL-E: Extended spectrum beta-lactamase producing Enterobacteriaceae
GH: Glycoside hydrolase
GT: Glycosyl transferase
KOs: KEGG orthologies
SCFA: Short chain fatty acids
