# Supplementary material for: Gut microbiome signatures of nursing home residents carrying Enterobacteria producing extended-spectrum β-lactamases
Source: Antimicrob Resist Infect Control. 2020 Jul 14;9:107. doi: 10.1186/s13756-020-00773-y (PMC7359458; doi:10.1186/s13756-020-00773-y)
Supplement: Supplementary file 1 — Additional file 1. Supplementary methods and figures. [file 13756_2020_773_MOESM1_ESM.docx]

Supplemental Material for

**Gut microbiome signatures of nursing home residents carrying Enterobacteria Producing Extended-spectrum β-lactamases**

Le Bastard Q^1,2^, Chapelet G^1,3^, Birgand G^1,4^, Hillmann B^5^, Javaudin F^1,2^, Hayatgheib N^1^, Bourigault C^1,6^, Bemer P^1,6^, De Decker L^1,3^, Lepelletier D^1,6^, Batard E^1,2^, Montassier E^1,2^

This file includes:

Supplementary methods: bioinformatic analysis, Supplemental figures 1 to 5, Captions for Additional data tables 1 to 5 (comma separated values .txt files)

**Supplemental methods: bioinformatic analysis**

**1. Features annotation**

Raw sequences alignment using BURST pipeline at 97% identity against the RefSeq database for taxonomical annotations. We included all matched bacterial species and all species in matched representative genera. For the functional characterization, we identified Kegg Orthologies using the HMAnN2 pipeline against the KEGG database. In detail, raw sequences were aligned against the UniRef90 database (v1.1) using HUMAnN2 and then translated against the KEGG database. Then, we focused on carbohydrate metabolism and annotated raw sequences against CAZy database using the blastx command of the DIAMOND sequence aligner. Biodiversity rarefactions, calculations and analysis (alpha and beta diversity) were computed using the QIIME2 diversity script. Comparisons were performed in R environment as described in the main manuscript.

**2. Identification of taxonomical and functional features characterizing the gut microbiome.**

As described in the main manuscript, we identified differences in abundance between samples from ESBL-E carriers and non-carriers using the DESeq2 package. Because we observed a low prevalence of ESBL-E colonization in our cohort, we decided to perform a bootstrap analysis in order to enhance statistical power and robustness of our analysis. Because of multiple testing, *P* values were adjusted using the Benjamini-Hochberg adjustment strategy to limit the occurrence of false positive candidates. As recommended in the literature, we performed 200 bootstrap analyses. Basically, for each run of DESeq2 analysis, the 10 ESBL-E positive samples were compared to 40 negative samples that were randomly selected. This sample representing a bootstrap sample.
**3. Identification of associations between clinical metadata and metagenomic data.**

MaAsLin is a multivariate statistical framework, part of the Biobakery environment. This script performs boosted additive general linear models between metadata (clinical data) and feature abundance. All features annotated (taxonomic and functional) were tested against baseline characteristics described in Table 1 plus location in the nursing homes (floor and building).

**Supplementary Figure 1. ESBL-E carriers and non-carriers harbors non-significantly different relative abundance of *K. pneumoniae, E. cloacae* and *E. coli.*** Individual plots of relative abundance of the three species identified as extended spectrum beta-lactamase producers in ESBL-E carriers. Comparison of relative abundances between ESBL-E carriers and non-carriers shows no significant differences (Mann-Whitney U test, ESBLE-E carriers versus non-carriers for E. coli, E. cloacae and K. pneumoniae relative abundances, all p values > 0.05)

**S1**

**Supplementary figure 2. ESBL-E carriers and non-carriers have a distinct gut microbial composition.** Beta-diversity comparisons of the gut microbiomes of extended spectrum beta-lactamase producing Enterobacteriaceae (ESBL-E) carriers and non-carriers. The first two axes are shown of principal coordinate analysis (PCoA) of Bray Curtis distances between individual communities at species level. The proportion of variance explained by each principal coordinate axis is denoted in the corresponding axis label. The plot shows a significant separation between samples from ESBL-E carriers and non-carriers (analysis of similarities, ANOSIM, R=0.177, p value=0.048).

**S2**

**Supplementary figure 3. Analysis of within samples functional biodiversity shows an increased biodiversity in the gut microbiome of ESBL-E carriers.** Alpha-diversity indices in gut microbiomes of extended spectrum beta-lactamase producing Enterobacteriaceae (ESBL-E) carriers and non-carriers., based on KEGG KO. Whiskers in the boxplot represent the range of minimum and maximum alpha diversity values within a population, excluding outliers. Monte-Carlo permutation t-test: *p value <0.05; ** p value <0.01; and *** p value <0.001. Boxplots denote top quartile, median, and bottom quartile.

**S3**

**Supplementary figure 4. KEGG KO that differentiate Enterobacteriaceae (ESBL-E) carriers and non-carriers at genus level, using DESeq2 with bootstrap iterations.** The bootstrap model consists in 200 random selections of non-carriers (n = 40). We represented the KEGG KO that are significantly different between ESBL-E carriers and non-carriers in more than 70% of the iterations, with a FDR corrected p value < 0.10.


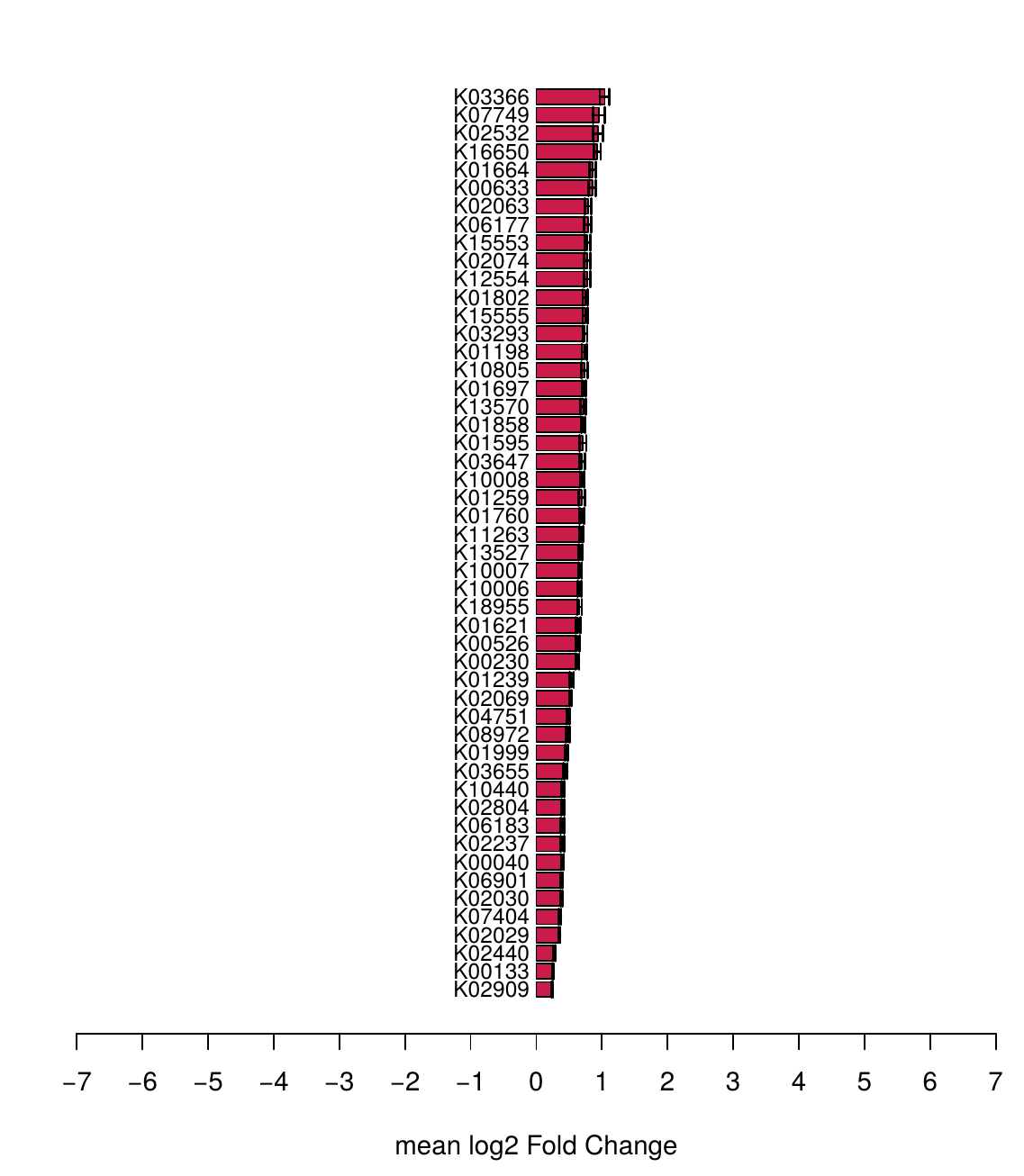

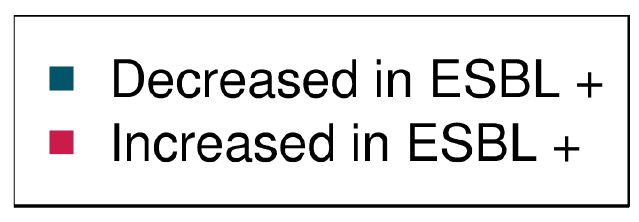

**Supplementary figure 5. CAZyme that differentiate Enterobacteriaceae (ESBL-E) carriers and non-carriers at genus level, using DESeq2 with bootstrap iterations.** The bootstrap model consists in 200 random selections of non-carriers (n = 40). We represented the CAZymes that are significantly different between ESBL-E carriers and non-carriers in more than 70% of the iterations, with a FDR corrected p value < 0.10.

**Supplementary table 1.** Genus that differentiate carriers and non-carriers using DESeq2 with bootstrap iterations.

**Supplementary table 2.** Species that differentiate carriers and non-carriers using DESeq2 with bootstrap iterations.

**Supplementary table 3.** KEGG modules that differentiate carriers and non-carriers using DESeq2 with bootstrap iterations.

**Supplementary table 4.** KEGG KO that differentiate carriers and non-carriers using DESeq2 with bootstrap iterations.

**Supplementary table 5.** CAZyme that differentiate carriers and non-carriers using DESeq2 with bootstrap iterations.
